# Power, danger, and secrecy—A socio-cultural examination of menstrual waste management in urban Malawi

**DOI:** 10.1371/journal.pone.0235339

**Published:** 2020-06-26

**Authors:** Heather Roxburgh, Kate Hampshire, Tamandani Kaliwo, Elizabeth A. Tilley, David M. Oliver, Richard S. Quilliam

**Affiliations:** 1 Biological and Environmental Sciences, University of Stirling, Stirling, United Kingdom; 2 Department of Anthropology, Durham University, Durham, United Kingdom; 3 Center for Water, Sanitation, Health and Appropriate Technology Development (WASHTED), University of Malawi (Polytechnic), Blantyre, Malawi; 4 Swiss Federal Institute of Aquatic Science and Technology (EAWAG), Dubendorf, Switzerland; Kyonggi University, REPUBLIC OF KOREA

## Abstract

Menstrual blood is not just a physical substance; it is laden with symbolism and often powerfully stigmatised. It is important to understand local perceptions and attitudes towards menstrual blood, as well as the preferred practices of menstruating women, in order to design appropriate sanitation and solid waste systems to support menstruation. Failure to take account of socio-cultural factors can jeopardise the effectiveness of such infrastructure. This study, conducted in Blantyre, Malawi, is a qualitative socio-cultural examination of how women manage and view menstruation. Thirty nine interviews, conducted with individuals and with small groups of friends, were carried out with thirty one women using pit latrines, flush toilets, and urine-diverting dry toilets in early 2019. Menstruation in Blantyre was found to be shrouded in secrecy because it was viewed as ‘dirty’, and therefore remained concealed. There was widespread anxiety about menstrual blood being used in *ufiti* (witchcraft), which affected how women used and disposed of their menstrual absorbents. At the same time, menstrual blood was also viewed as a powerful healing substance with uses in traditional medicine. The type of infrastructure required by women to support their menstruation depended on the type of menstrual absorbent used. Those using reusable cloth generally preferred a private bathroom with discreet drainage, whilst those using disposable pads needed a discreet and convenient disposal system. Increased preference for disposable pads over reusable cloth (particularly for younger women in education or employment) suggested that menstrual waste profiles of urban areas may be changing. Understanding these changing needs will be crucial for planning effective, sustainable waste disposal and sanitation infrastructure.

## 1 Introduction

Menstruation and menstrual blood are not just physiological or physical phenomena; they are steeped in symbolism and subjectivity [1, 2]. Globally, there are substantial spatial and temporal variations in how menstruation is regarded and managed, and how menstruating women are expected to behave [3]. For example, in particular contexts, menstruation can affect women’s participation in religious life [4, 5] and can cause a degree of withdrawal from public life, ranging from the avoidance of crowded places [6] to total isolation [7]. Some menstruating women may be expected to abstain from cooking, washing, or household chores [3, 7], whilst others do not refrain from anything at all [8].

Some broad trends are associated with the social conceptualisation of menstruation across different contexts. One common framing is of a “dirty”, “polluting” process [3, 6], under which menstruation and menstrual blood are stigmatised [9]. As a result of such stigmatisation, many women around the world feel obliged to keep their menstruation secret and conceal any visible signs of bleeding [3, 6, 7]. Whilst this stigmatisation is challenged in some spaces and spheres [10], it remains common in many parts of the world.

Menstruation, and the associated term ‘menstrual hygiene management’ (MHM), have become increasingly recognised in the water, sanitation and hygiene (WASH) development sector as an important and gendered aspect of sanitation and health [11, 12]. Infrastructural services that provide waste disposal and/or privacy—including water supply, sanitation and bathroom facilities, and solid waste collection—all play into women’s experiences and practices of menstruation [13]. However, as Sommer et al. [14] and others have argued, this infrastructure must reflect local norms and preferences around menstruation, rather than being merely replicated in different contexts [14–16].

Failing to take account of locally-specific ways in which menstruation is understood and managed can fundamentally undermine the effectiveness of health and environmental interventions. For instance, trials of a HIV-prevention vaginal ring in southern and central Africa showed that women preferred to remove their rings during menses, because of concern that the rings would impede the flow of blood and affect bodily hygiene; this compromised effectiveness of protection against HIV [17]. Similarly, a preference towards disposal of menstrual waste in pit latrines can hamper the economic viability of the pit emptying sector, and impede the recovery of value from faecal sludge in the form of agricultural nutrients or energy [14, 18, 19].

The objective of this study was to examine, qualitatively, practices, preferences, and socio-cultural norms surrounding menstruation among women using three types of sanitation facility in Blantyre, Malawi–pit latrines, flush toilets, and urine-diverting dry toilets (UDDTs). Pit latrines are the most common type of toilet facility in Blantyre, used by 78% of the population, followed by flush toilets (21%) [20]. UDDTs are relatively uncommon, used by less than 1% of the population, but are promoted in Blantyre and more widely across Africa by advocates of ecological sanitation. These are a type of resource-recovery toilet that separates urine and faeces at the point of use by diverting urine into a tank or into the ground and allowing faeces to fall into a dehydration vault. Water and solid waste should not enter the faecal vault of a UDDT as this can compromise the dehydration process [21]. The particular ways in which these three sanitation facilities facilitate or hinder management of menstruation are examined, with the aim of providing insights into improved service design.

## 2 Methods

Fieldwork was carried out in early 2019, and involved conversational interviews with 31 female participants between the ages of 19 and 63, living in the city of Blantyre, Malawi. Most of these women (N = 23) were interviewed individually (35 interviews in total, as some participants were interviewed more than once). A small number (N = 9) of participants were interviewed in small groups (N = 4). In addition, two Malawian female key informants (KIs), colleagues at the University of Malawi, provided contextual information over the course of on-going discussions. Initially, sampling was restricted to women who had not yet reached menopause and therefore had recent experience of menstruation to discuss with the researchers. However, this was later expanded to include post-menopausal women who could provide perspective on certain issues of interest, such as changes in societal openness around discussion of menstruation.

Participants were recruited from two locations, chosen to represent contrasting provision of sanitation facilities. Twenty women were recruited from a low-to-middle income suburb of Blantyre, where households typically used either pit latrines or UDDTs (promoted and installed by a local NGO). Recruitment in that site was achieved by following a transect walk across the suburb, and approaching a house every 50m to explain the purpose of the study and invite residents to participate. If the household was not interested, did not contain a woman over 18, or nobody was at home, then the next house was approached. The second recruitment location was a university campus, where 11 users of flush toilets and pit latrines were recruited by standing at a central point on the university campus and inviting passers-by to take part, and also by snowball sampling, whereby participants were asked to recommend others who might be willing to participate. Participants from the campus were a mix of female students and campus staff (cleaning and security). Refusal to participate was around 30%, with the most commonly cited explanation being unwillingness to spend time, or not seeing any personal benefit from participating. In addition, further insight was obtained through discussion of the research with the KIs, which induced contributions of their own personal experiences and reflections on the topic. With their permission, notes from these conversations were analysed alongside the interviews.

Most participants were interviewed individually; however, in some cases participants were with a female friend or relative when the interviewer arrived, and requested that the interview be carried out with them present. In other cases a female friend joined them partway through the interview, and the participant requested that the interview continue, with the friend also contributing responses and thus creating a relaxed and sociable feeling (friends and family who sat in on the interviews were not formally counted as part of the 32 participants, and their details are not included in Table 1). Subsequently, this atmosphere was therefore encouraged further through the recruitment of friends for small group interviews (2–3 participants). Interviews were initially conducted through a set of open-ended questions (e.g. what menstrual absorbents do you use, and how do you dispose of them?), but eventually moved towards a guided conversation format (e.g. please tell us about how you manage your menstruation), which encouraged participants to explain, explore and discuss different topics that had emerged from the data as well as describing their own experiences. Follow-up interviews were conducted where clarification was needed, or when the researchers had further questions after reflecting on the interview. These were mostly conducted with participants interviewed early on, as the volume of new insights decreased throughout the data collection process. Participants were also asked to reflect on preliminary conclusions and offer opinions on their validity.

**Table 1:** Characteristics of participants.

Characteristic	No (%) participants
*Age group*	
19–29	14 (45%)
30–39	6 (19%)
40–49	5 (16%)
50–59	4 (13%)
60+	1 (3%)
Not known	1 (3%)
*Menstrual absorbent*	
Cloths	13 (42%)
Pads	13 (42%)
Cotton	2 (6%)
Tampons	1 (3%)
Not known	2 (6%)
*Toilet used at home*	
Pit latrine	7 (23%)
UDDT	11 (35%)
Flush	6 (19%)
Not known	7 (23%)
*Recruitment location*	
Blantyre suburb	20 (65%)
Institutional campus (staff)	5 (16%)
Institutional campus (student)	6 (19%)

Interviews were conducted by two of the authors (Tamandani–a Malawian researcher at the University of Malawi, and Heather–a British PhD student). Individual and group interviews were conducted in the preferred language of the participant, which was usually Chichewa (two were conducted in English). All but one participant agreed for their interviews to be recorded; these were then transcribed, and (for interviews in Chichewa) translated to English. For the one unrecorded interview, notes of the conversation were made immediately afterwards. Each transcript with associated notes was then read and discussed line by line by the two interviewers, to ensure a commonality of understanding and interpretation. Where transcripts contained ambiguity, participants were contacted again to clarify the meaning of their comments. Analysis was conducted by inductive thematic coding, based on the principles of grounded theory [22]. The two interviewers (Heather and Tamandani) read transcripts and notes closely, and then noted and compared emerging impressions and themes. Subsequently, one author (Heather) developed a series of inductive codes, and the transcripts coded in Nvivo (version 12, QSR International). The codes were based around two main analytical categories, with some degree of overlap: codes relating to socio-culturally constructed beliefs, attitudes and practices surrounding menstruation, and codes relating to the physicality and practicality of menstruation’s interaction with sanitation and solid waste management infrastructure. Below, pseudonyms are used, and an age range (rather than exact age) is shown to preserve anonymity.

Ethical consent was obtained from the University of Stirling General University Ethics Board before commencement of fieldwork. All participants provided informed, written consent to partake in the study.

## 3 Results

Characteristics of participants are shown in Table 1.

### 3.1 Menstrual absorbents used

The two most common menstrual absorbents used by participants were disposable shop-bought sanitary pads (42%), and menstrual cloths (42%), the latter made from pieces of old clothing or blankets, used to absorb blood, and then washed, dried, and reused. In addition, two women used cotton wool and gauze to fashion home-made sanitary pads, locally referred to as ‘cotton’. Only one university student used tampons on an occasional basis, and so these are not discussed further. As the usage of shop-bought and home-made pads are similar in many regards, they are discussed under a single term–pads.

### 3.2 Cultural aspects of menstruation

#### 3.2.1 Secrecy and respectability

All participants described a profound secrecy around menstruation, as Chifundo [50s, suburb, UDDT, cloth] explained: ‘*It’s because of our culture*, *it doesn’t allow that anyone should see [blood]’*. It was important for all participants to hide any visual, olfactory, or behavioural signs of menstruation, and they would exert considerable effort and energy towards concealment. It was considered shameful for others to see signs of menstruation, as Elizabeth [late teens, suburb, pit latrine, pads] described: ‘*When I use the toilet*, *maybe I have spilt some blood and I didn’t check and see it*, *and my dad enters the same toilet*, *he may feel disgusted… it doesn’t bring respect’*. It was considered particularly essential to keep the signs of menstruation secret from men and young children; some participants even described it as ‘dangerous’ for them to see signs of menstrual blood. Others spoke about hygienic and moral imperatives to keep menstruation secret. Pilirani [20s, KI, flush, pads] described a conversation she had with her mother, who told her: ‘*Menstrual blood is filthy*. *It is not like the regular human blood*. *It stinks*, *so for others to see it is not good at all’*. Two participants mentioned that people may discreetly refuse to eat food that they had prepared if they were known to be menstruating: ‘*Others when you cook food may say they are full*, *they don’t want the food*, *and see you as filthy’* [Dalitso, 20s, campus-student, flush, pads]. Most women said they would feel comfortable discussing menstrual health concerns with a doctor, due to their perceived confidentiality. Chifundo [50s, suburb, UDDT, cloth] said: ‘*Menstruation is a private thing… so if you have any problems it’s better to go and see a doctor who cannot disclose it to anyone’*. Some women commented that secrecy around menstruation was less severe than previously, with young women today feeling more freedom to share their menstrual problems with friends, and in trusted spaces like church groups.

#### 3.2.2 Menstrual cloths as healing objects

In apparent contrast to the taboos associated with menstrual blood described above, many women also believed that menstrual blood had healing properties, as Fatsani [30s, suburb, pit latrine, cloth] commented: ‘*There is goodness in menstrual blood*’. Small amounts of menstrual blood remaining in the menstrual cloth after washing (as evidenced by staining and discolouration of the material) was believed to give the cloth medicinal qualities; cloth which had never been used to absorb menses, or ordinary blood, were not thought to have the same effect. Chisomo [50s, campus-staff, cloth] explained ‘*The thing making cloth to be powerful is the [menstrual] blood*.*’* Many participants, particularly those who were older, described using (or being aware of others using) cleaned menstrual cloth in traditional medicine practices. Kondwani [40s, suburb, pit latrine, cloth] explained the use and process: *‘If your child gets sick then you take the [clean] menstrual cloth and warm it on the fire then put it on the body of the baby and the baby gets well*, *or even when you are having flu or cough*, *you take the menstrual cloth and warm it on the fire*, *then inhaling it the cough or flu goes away*.*’* Limbani [50s, suburb, UDDT, cloth] added another example: ‘*If your child has been burned by fire*, *we take [the menstrual cloth] and we use it on the wound*, *and the wound dries up so easy’*. Several women kept their menstrual cloths in case they were required by an ill or injured family member, even after they were worn out and no longer functional in their original sense. One woman required permission from her husband before discarding menstrual cloths, as they were considered so valuable.

#### 3.2.3 Fear of *ufiti*

Almost every participant feared menstrual blood being used in *ufiti* (witchcraft). If another person got hold of a menstrual cloth or used pad, or even stained underwear, it was believed that they could take it to a *sing’anga* (witch doctor) and use it to cast *ufiti* over the owner of the object, causing them harm. As Mercy [30s, suburb, UDDT, cloth] explained, ‘*It is dangerous for us women*, *taking the cloth with blood and disposing it… Someone sees it*, *maybe they can do some bad things to you*’. Possible effects were said to include menstruating continuously for several months at a time, infertility, or (more rarely) death. Many women, old and young, claimed to know friends or family members who had personally experienced harm from *ufiti* through stolen menstrual absorbents, and it was perceived as a relatively common occurrence. Mayeso [30s, suburb, pit latrine, cloth] described the experience of her mother: ‘*Other people can make you to be barren*, *like my mother*, *her cloth was stolen and she couldn’t conceive again*.*’* Some women also believed that menstrual cloth or used pads could be stolen and used in other kinds of *ufiti* to bring wealth or prosperity to the thief.

The secrecy around menstruation was therefore due not only to embarrassment, but also to fear. Women took great care to keep their menstrual cloths secure and hidden at home, so that they were not vulnerable to *ufiti*. Some spoke of taking pains to hide them from their own husbands. For those who used disposable pads, they took care to dispose of these ‘safely’ so that nobody else could get them. Elizabeth [late teens, suburb, pit latrine, pads] described how she protected her used pads from being used in *ufiti*’: *‘it’s just to be storing them safely like I do*, *I store them [in the bedroom] then burn them*, *in order to avoid such kinds of things’*. Whilst burning and discarding in pit latrines were thought to be safe options, many women were wary of the idea of discarding their pads in the open, or leaving them in a bin. The higher volume of waste created as a result of using disposable pads was seen as a negative aspect by some women, who felt that cloth was safer and left them less at risk of *ufiti*.

Fears of *ufiti* was generally felt more acutely by the older women. Some (but not all) of the younger university-attending women were prepared to discard pads in the bathroom bins on campus, provided that the bathrooms were kept locked out of hours, which they felt provided adequate protection against *ufiti*. The older women confessed their fears for the safety of the younger women as a result of their more relaxed attitudes; Tadala [40s, campus-staff, cloth] gently admonished the young female interviewer: *‘You younger generation*, *you don’t take things seriously*, *you don’t even think that your own [pads] can affect you’*. Some, however, felt that times had changed and the risks of *ufiti* had reduced; Takondwa [60s, suburb, cloth] commented: *‘During the olden days it was more dangerous [than now]’*. Chimwemwe [30s, suburb, UDDT, pads] regretted seeing more discarded waste as a result of this attitudinal shift: *‘Pads*, *they are not well taken care of*, *they are just everywhere*, *because now people are not afraid of blood any more’*. Mphatso [30s, campus-staff, cloth] reflected on the differences, and speculated that it might be due to education: ‘*Our parents*, *when they teach us that*, *my child*, *take care of [the cloth] when menstruating*, *we were listening*, *maybe because of being uneducated*. *But now*, *today*, *when you tell them that cloths*, *don’t keep them anywhere… they see it as useless*.*’*

#### 3.2.4 Menstrual restrictions

There were a number of restrictions associated with the state of menstruation. Acts mentioned by participants which should be refrained from during menstruation included: adding salt to food; having sexual intercourse; picking vegetables from the field; having physical contact with children; and cooking food. The most common abstinence discussed was adding salt to food; this was something that several participants were advised to do by their elders, although none were able to explain why. These women would typically ask another member of the family to add it instead. The salt restriction appeared to be viewed similarly to a religious affiliation, with some women choosing to adhere to it and others choosing not to. Tadala [40s, campus-staff, cloth] explained: ‘*Others say [when menstruating] that you shouldn’t add salt to relish [a dish of vegetables and/or meat to accompany rice or nsima]*, *but me*, *with the way I pray*, *I don’t see that there is any problem with it much*.*’* Some of those who refrained from adding salt added that whatever danger was once associated with it may no longer be present, but they still prefer to keep the practice anyway. Limbani [50s, suburb, UDDT, cloth] explained: *‘The way we were told*, *when menstruating*, *adding salt*, *people [who eat the food] get swollen*. *But now*, *it’s no longer there*, *we can add salt*. *But to us who got adapted [to not adding salt]*, *we are still believing that*.*’*

### 3.3 Practical aspects of menstruation

Both pads and cloth require eventual disposal; pads are disposed after each use, and cloth is ultimately disposed when the material is worn out through repeated washing and no longer absorbs liquid. Pads generate a considerably larger volume of menstrual waste than cloth, which is often reused for months or years. Most participants disposed of their pads and cloth either by burning them, or by discarding in pit latrines. Methods of disposal were more complex outside of the home; some participants would dispose of their pads in bins at their place of work or study if they needed to change their pad during the day, whilst others would carry their used pads home to burn them there. Other participants, who were not comfortable with the disposal options available to them at home, would carry all of their used pads to their place of work or study for discarding. Women would often use a combination of disposal methods according to what was convenient at the time.

#### 3.3.1 Reuse by washing

In terms of day-to-day menstruation management for cloth-using women, the most important household facility was the bathroom. In many Malawian households, the bathroom is located in a separate building to the main dwelling, and is typically a small room with a drain leading into either: the pit latrine; the ground (i.e. an improvised soakaway); or an open channel, possibly connecting to a public ditch or discharging elsewhere in the compound. The bathroom is usually separate to the toilet room, but may be adjacent. All washing activities (of the body and the cloth) take place in the bathroom, and the privacy of the space is very important. Water draining from the bathroom should not be publically visible, so that bloodied water from washing the cloth cannot be seen by neighbours or passers-by. One cloth-using participant, Fatsani [30s, suburb, pit latrine, cloth], commented that covered drainage from her bathroom was the thing she desired most in her whole life.

#### 3.3.2 Disposal by burning

Disposing of pads and cloth by burning was preferred by many women but, owing to the secrecy around menstruation, this had to be carried out in a discreet location either early in the morning or in the evening when nobody else was around. Pilirani [20s, KI, flush, pads] explained: ‘*Girls are supposed to… burn them in a secret place*, *if there are men in the house you’re supposed to wait for them to be out*.’ Some women noted that it can take a long time for a pad or cloth burn completely, particularly if it is wet. In order to speed up the burning process, they would therefore sometimes leave pads to dry in the sunshine on toilet rooftops, but admitted that they worried about the pads being stolen and used for *ufiti* whilst left unattended. Others mentioned that burning pads causes a noticeable odour, and which caused them to feel uncomfortable as it could alert neighbours to their activity: ‘*When you burn*, *it will produce air pollution*, *and people around the neighbouring houses will notice something [i*.*e*. *that you are menstruating]’* [Thoko, 20s, campus-student, flush, pads]. Despite these inconveniences, burning was generally regarded as a safe solution as it turned the pads and cloth to ash, protecting them from being used in *ufiti*, and preventing them from being seen by anybody. In one unusual case, a woman burned her pads directly inside her UDDT vault, by burying them in hot ash.

In order to conserve matches and paraffin, some women preferred to burn their used pads together, in one go, at the end of their period. However, storing used pads discreetly was challenging as they emitted a strong odour and attracted flies. Women worried about other people in the household smelling or finding the pungent pads, as well as experiencing personal discomfort from keeping them in their bedrooms. Pilirani [20s, KI, flush, pads] explained how she would ‘*gather [the used pads] in a plastic bag under my bed*, *they produce some bad smells and so windows are to be open*, *but during the night*, *eeiish…’* Having to store odorous pads in a bedroom shared with young children or boys caused particular anxiety. Some, however, developed innovations: Margaret [late teens, suburb, UDDT, pads] stored her used pads in an airtight container (made from an old paint tub) in her bedroom, which effectively contained the odour, after hearing the idea from a friend at church.

#### 3.3.3 Disposal in toilets

Throwing pads and cloth in pit latrines was another common disposal route. This was regarded as a particularly useful ‘emergency’ option, as items could be dropped into a pit latrine quickly and discreetly. However, some pad-using women preferred not to routinely discard pads in their own pit latrines as they feared that it would cause the pit to rapidly fill up, shortening its useful lifespan, and would therefore only throw the occasional pad or cloth when unable to dispose of it by burning. If the pit latrine was designed for routine emptying, some women also had concerns about discarding pads in these toilets because of the potential for pads to physically cause problems with emptying devices (described by Elizabeth [late teens, suburb, pit latrine, pads] as ‘*blocking the pipes’*), and also due to embarrassment from others seeing the pads during the emptying process.

Those with flush toilets or UDDTs, unable to throw pads or rags in their own toilets, would sometimes secretively throw their items into their neighbour’s pit latrines. Tiyamike [20s, suburb, UDDT, pads & cloth], who had a UDDT, admitted: ‘*Sometimes you fail to wash menstrual cloth*, *then you say I should dispose to the neighbour’s toilet [a pit latrine]… you are to wait for the neighbour to be out or not around*, *then you run and dispose them*.’ However Tiyamike worried that this could cause conflict with her neighbours if her actions ever came to light.

#### 3.3.4 Disposal in bins or dumps

Discarding pads or cloth into the solid waste system, whether in bins or on local dumpsites, was generally seen as an unfavourable option and used as a last resort. This was partly due to the risk of pads or rags being taken (from either the bin, or the final destination) and used in *ufiti*, but also because it contravened the expectation that they should be kept hidden, and was therefore seen as immoral and disrespectful. This feeling was particularly strong among the older generation of participants, who felt dismayed to see bloodied pads scattered around like litter. Younger pad-using participants expressed particular anxiety about dogs scavenging their pads from bins and carrying them away, which may account for how pads end up dispersed in the urban environment despite the general wish to keep them hidden.

In spite of the general discomfort around discarding pads in bins (due to not knowing the ultimate destination of the waste, and whether this would be safe from *ufiti*), some campus students who had flush toilets at home and did not wish to burn their pads would nonetheless carry them to university each day and leave them in the bins there. Joyce [20s, campus-student, flush, pads], who has a flush toilet at home, explained: ‘*I don’t feel comfortable burning them*, *so most of the times I do carry the ones that I’ve used*, *and throw them in the bin at the [campus] toilet*.’ Cleaners at the campus confirmed that they regularly find bundles of used pads in plastic bags discarded in the bins.

## 4 Discussion

This study has provided detailed insights into the ways that different sanitation facilities in a low-resource setting can affect the management of menstruation, with important implications for infrastructural planning. The qualitative interview approach has provided rich data that offer a more nuanced understanding of how cultural beliefs, material constraints, and practical considerations can intersect, against a background of generational and socio-economic change, to shape women’s experiences and practices of menstruation. Based on a sample of just 31 women in one city, the study cannot claim to provide a generalizable or representative picture of menstrual practices and beliefs in Blantyre or beyond; however, the ubiquity of certain themes, practices and beliefs described by participants suggests that these might be widely held, which could be confirmed by further research. The interviews revealed themes, beliefs and practices with relevance to sanitation and solid waste management infrastructure design, which are discussed in turn below.

### 4.1 Concealment in plain sight

The characterisation of menstruation as ‘unclean’ and ‘shameful’ concurs with similar studies elsewhere in sub-Saharan Africa [23, 24], and is akin to many other cultures around the world [1, 9]. This leads to a culture of total concealment around menstruation. Whilst men are generally aware that women of reproductive age menstruate monthly, efforts to mask it essentially erase the status from public view. This aligns with Foucault’s concept of ‘heterotopias of deviation’, whereby undesirable bodies are sequestered in particular, private places, such as psychiatric hospitals and prisons, in order to preserve a public ‘utopia’ [25]. In the case of menstruation, any detectable signs are dealt with in strict privacy in order to preserve the public-facing facade of clean, hygienic, non-menstruating women. This has led to ‘culturally sanitised’ versions of women becoming normalised, whilst those defying or not fitting the norm may be stigmatised [26]. Indeed, Bobel [27] argues that even the use of the word ‘management’ in MHM–a period-positive movement—implies that the body is unruly in its natural state, requiring ‘protection’ through ‘sanitary’ products.

Recognition of the illusion of female non-menstruation, and whether/how to challenge menstruation stigma, inspires continued debate. Bobel [27] suggests that by centring the mission of MHM on providing women with privacy and materials to conceal their menstruating status, MHM actually perpetuates the stigma surrounding menstruation rather than challenging it. However, it is not necessarily contradictory to challenge menstrual stigma whilst also supporting women’s access to privacy for the actual act of cleaning and/or discarding menstrual materials and blood, if desired.

Despite the aura of secrecy, in this study it was possible to speak openly about menstruation with women, and the interviews were conducted with ease. Whilst many participants said that they would not routinely discuss menstruation with their friends, others did not mind the presence of their friends during interviews, and interviews with small groups of friends were found to create a relaxed atmosphere. The general level of secrecy around menstruation was perceived by many women to be decreasing, which has been noted in similar studies elsewhere [3]. Whilst the severe stigma attached to the sight of blood or bloodied materials is certain to persist for now, participants appeared to feel able to speak more freely about these topics than in decades past.

### 4.2 Paradox of dirt and value

Menstrual blood was perceived to be a powerful substance, with great potential for good or evil. In common with the Gambia [6], the fear of *ufiti* cast by *sang’anga* had a significant influence over how women handled and disposed of their menstrual waste, and even (for some) their menstrual absorbent preferences. These fears lie within a Malawian context of widespread and acute belief in *ufiti*, which is found both urban and rural areas, and periodically causes outbreaks of national panic [28]. Use of menstrual cloth in healing practices, however, has not been identified elsewhere in the literature, either in Malawi or sub-Saharan Africa; it would be interesting to find out how widely such beliefs and practices might pertain.

There would appear to be a paradox between the conceptualisation of menstrual blood as filthy, disgusting, shameful, and dangerous, whilst being simultaneously revered for healing. Drawing on theories of dirt and disgust can help to explain this apparent contradiction. Douglas’ conception of ‘dirt’ as a substance or object that subverts an established cultural order [29] resonates deeply with menstrual blood, which is a substance that has come out of its usual place (circulating within the body), to transcend bodily boundaries. Meanwhile, the more naturalistic theory of disgust proposed by Curtis characterises revulsion as an evolved psychological system designed to help us avoid potential sources of disease [30]. Encountering substances secreted or excreted from other human bodies, such as menstrual blood, naturally evokes a response of disgust as they induce undesired intimacy [31]. Thus, menstrual blood is essentially ‘dirty’ on several counts. However, once menstrual blood has been washed, dried, and transformed into an odourless mark on a cloth, the automatic disgust triggered by the sight of a vivid human secretion is tempered. This allows another attribute to emerge: rule-breaking objects such as menstrual blood can be believed to embody great power, with both positive and negative potential [2, 29]. Uses of menstrual blood in protective charms and pre-scientific medicine (e.g. in Ancient Rome and Morocco) demonstrate these positive and negative poles of symbolic menstrual power [2]. Similarly, menstrual blood is also conceived of as a symbol of fertility and new life, despite being simultaneously seen as polluting [4, 32]. Menstrual symbolism is therefore highly complex, and can mirror that of the post-menarche female body in general: concurrently reviled and revered, viewed as polluting but also life-giving, and requiring regulation and control [5].

### 4.3 Menstrual absorbent type matters

When considering menstruation, it is critical to appreciate that the types of infrastructure and services required by women will vary depending on the type of menstrual absorbent that they use. Women who use cloth require a private bathroom to wash them in, with discreet drainage so that bloodied washing water is not visible (for instance, draining into a pipe or pit latrine, rather than discharging into an open drain that can be seen by neighbours). Another requirement is a private place to dry and store cloth, which was generally reported as the bedroom. By contrast, the toilet itself may be less relevant. During interviews, many cloth-using women seemed puzzled to be questioned about their toilet in relation to menstruation; this facility was not relevant to their menstrual management activities. Whilst a pit latrine (as opposed to other kinds of toilet) could be useful to occasionally dispose of a cloth, having discreet bathroom drainage was seen as considerably more important. The importance of discreet drainage from washing spaces has been described similarly elsewhere, for instance in the design of bathrooms in refugee camps in Pakistan [33].

For those who use pads, the most pressing need is for an inconspicuous disposal system. Women who feel comfortable burning their pads require a discreet place to do so, along with a supply of matches and paraffin, and should be made aware that fumes from the burning pads can contain carcinogens formed from chlorine [18]. Pit latrines were widely appreciated as a disposal method due to convenience and discretion, so, ironically, more sophisticated sanitation facilities that cannot receive pads (such as UDDTs, flush toilets, or pit latrines which are regularly emptied) may be less convenient for pad-using women if they do not have an alternative suitable waste disposal system.

MHM can therefore be seen to sit between the two distinct sectors of WASH and solid waste management, interacting with systems on either side, depending on what menstrual absorbent is used. As a result, whilst linkages between MHM and WASH have been correctly highlighted, these have sometimes been misleadingly characterised through an overemphasis on the role of toilets at the household level, when actually their relevance may depend on whether a woman uses disposable or reusable menstrual absorbents. For example, one study considered how national water and sanitation coverage estimates might be used to identify barriers to MHM and suggested that data on open defecation may provide a useful proxy [34]. Whilst the authors acknowledge limitations of their indicators, the most crucial of these is not fully discussed: women using cloth do not necessarily manage their menstruation in the toilet, and openly defecating women are likely to be poor, and therefore likely to be cloth-users. Thus, open defecation rates, or quality of toilet facilities in general, are unlikely to provide a reflection of the state of menstrual hygiene management. Others have studied the relationship between household sanitation and experiences of menstruation in Nigeria, but did not look specifically at household bathrooms [35]. The lack of relevance of toilets may explain why they were unable to find any meaningful relationship, and concluded that ‘existing [sanitation] indicators are not suitable to women’s menstrual needs’.

### 4.4 Changing composition of menstrual waste

Disposable sanitary pads have become much more widely available in the last two decades, and are now commonly used (particularly by younger women) in urban areas. Many younger women interviewed felt disgusted by the idea of washing and reusing cloths, and feared being teased by their peers for using them. However, despite appreciating the convenience of disposable pads, many pad-using participants found disposing of their pads discreetly to be inconvenient, time and labour intensive, and stressful.

Given the increasing preference of younger and more educated women for using pads, it would appear that the menstrual waste profile of urban Malawi is changing rapidly. Increasing preference for disposable pads over reusable cloth will mean a dramatic increase in the volume of menstrual waste produced. This trend is unlikely to be associated with the poorest women, who will probably continue using cloth due to their lower cost, and will be most pronounced in middle income groups, and among women in education or employment (due to the convenience that disposable pads offer for moving around). Traditional methods of disposal, such as burning or discarding waste in pit latrines, may come under pressure as a result of the increased volume of waste associated with pad use.

### 4.5 Future of menstrual waste management

Menstrual waste disposal issues resulting from a combination of socio-cultural constraints and inadequate infrastructure have been reported in this study, and elsewhere around the world [18, 36]. Participants described important requirements around the management of menstrual waste (i.e. that it is unseen, and secure from use in *ufiti*) that result in methods of disposal being used that can have negative effects on health, sanitation, and the environment. For instance, burning can expose users to carcinogenic fumes, whilst disposal in pit latrines results in rapid filling and emptying problems. These challenges are particularly pertinent for disposable pad users, who generate greater volumes of waste. When the rapidly increasing popularity of disposable pads is also considered, it is clear that environmental and public health consequences could be serious if suitable infrastructure, products, and services, are not made available to women.

However, in order to see the way forwards for menstrual waste management, there now needs to be an expansion and integration of qualitative understanding with quantitative data on urban menstrual waste generation and disposal in order to confirm significance and extent. Initiatives around solution design, concerning both waste generation and disposal, require a strong participatory component to ensure suitability for women's needs and preferences. For instance, participatory research could explore whether community menstrual waste disposal points, or waste storage facilities at the household level, can be innovatively designed in such a way as to satisfy user requirements for privacy, security, and hygiene. Meanwhile, in order to reduce the generation of menstrual waste, further research could explore how to improve the availability and manufacturing standards of high-quality reusable sanitary pads. These combine many of the beneficial aspects of disposable pads (e.g. high absorbency, comfort), with traditional practices (i.e. washing and reusing cloth, which generates smaller volumes of waste), but also improve on them by utilising materials with fast drying times and requiring small volumes of water for washing [37, 38]. Development and promotion of affordable compostable pads could potentially make disposal in pit latrines more compatible with pit emptying programmes, although this has admittedly proved a challenging venture elsewhere [27, 39].

## 5 Conclusions

Using qualitative interview methods to understand practices, preferences, and socio-cultural norms surrounding menstruation among pit latrine, flush toilet, and UDDT users in Blantyre has revealed that: i) menstrual blood is a highly sensitive substance, culturally imbibed with significant power for harm and healing; ii) the kinds of menstrual absorbents that women use dictate their infrastructural and service needs; and iii) increasing preference for disposable pads over reusable cloth suggests that Blantyre’s menstrual waste profile may be changing substantially. Menstrual waste is no ordinary waste: the complete concealment of blood, and the state of menstruation in general, is important due to shame, stigmatisation and fear of blood being used in *ufiti*.

Socio-cultural norms must be taken into account in order for the infrastructure to be used effectively, and these might not be immediately obvious to service designers (especially those who are male). As a result, there is a strong case for the use of participatory methods to develop user-designed menstrual waste management solutions. Crucially, pad users and cloth users must be recognised to have different needs and priorities. Our research suggests that for pad users, solutions might be centred on discreet and convenient disposal systems which are not easily accessible to others, whilst for cloth users there might be a greater focus on private spaces with covered drainage to wash and dry cloths.

Other avenues that would benefit from further research include characterising Blantyre’s menstrual waste profile, i.e. quantifying the volumes produced of different types of menstrual waste and identifying their destinations. This would allow the potential benefits of capturing menstrual waste on both environmental and human health to be evaluated, in addition to providing data for the design of solid waste management services. Our research suggests increasing preferences for disposable sanitary pads among younger women, indicating that the overall volume of menstrual waste is likely to be growing rapidly in line with demographic change.
